# Quantitative lung morphology: semi-automated measurement of mean linear intercept

**DOI:** 10.1186/s12890-019-0915-6

**Published:** 2019-11-09

**Authors:** George Crowley, Sophia Kwon, Erin J. Caraher, Syed Hissam Haider, Rachel Lam, Prag Batra, Daniel Melles, Mengling Liu, Anna Nolan

**Affiliations:** 1 Department of Medicine, Division of Pulmonary, Critical Care and Sleep Medicine, New York University School of Medicine, New York, NY USA; 2 Fire Department of New York, Bureau of Health Services and Office of Medical Affairs, Brooklyn, NY USA; 3 New York University School of Medicine, New York, NY USA; 4 University of California, Berkeley, Berkeley, CA USA; 5 Department of Environmental Medicine, New York University School of Medicine, New York, NY USA; 6 Department of Population Health, Division of Biostatistics, New York University School of Medicine, New York, NY USA

**Keywords:** Emphysema, Obstructive airways disease, Lung architecture

## Abstract

**Background:**

Quantifying morphologic changes is critical to our understanding of the pathophysiology of the lung. Mean linear intercept (MLI) measures are important in the assessment of clinically relevant pathology, such as emphysema. However, qualitative measures are prone to error and bias, while quantitative methods such as mean linear intercept (MLI) are manually time consuming. Furthermore, a fully automated, reliable method of assessment is nontrivial and resource-intensive.

**Methods:**

We propose a semi-automated method to quantify MLI that does not require specialized computer knowledge and uses a free, open-source image-processor (Fiji). We tested the method with a computer-generated, idealized dataset, derived an MLI usage guide, and successfully applied this method to a murine model of particulate matter (PM) exposure. Fields of randomly placed, uniform-radius circles were analyzed. Optimal numbers of chords to assess based on MLI were found via receiver-operator-characteristic (ROC)-area under the curve (AUC) analysis. Intraclass correlation coefficient (ICC) measured reliability.

**Results:**

We demonstrate high accuracy (AUC_ROC_ > 0.8 for MLI_actual_ > 63.83 pixels) and excellent reliability (ICC = 0.9998, *p* < 0.0001). We provide a guide to optimize the number of chords to sample based on MLI. Processing time was 0.03 s/image. We showed elevated MLI in PM-exposed mice compared to PBS-exposed controls. We have also provided the macros that were used and have made an ImageJ plugin available free for academic research use at https://med.nyu.edu/nolanlab.

**Conclusions:**

Our semi-automated method is reliable, equally fast as fully automated methods, and uses free, open-source software. Additionally, we quantified the optimal number of chords that should be measured per lung field.

**Electronic supplementary material:**

The online version of this article (10.1186/s12890-019-0915-6) contains supplementary material, which is available to authorized users.

## Background

Environmental exposures due to ambient particulate matter (PM), tobacco products or other occupational exposures may cause morphologic changes in lung parenchyma. Methods to quantify these structural changes have been an area of investigation spanning several decades [1–5]. These measures are important since they reflect clinically significant structural changes that can be a focus of therapeutic assessment [1, 2]. Mean linear intercept (MLI) is a measure of morphometric change based on serial measurements of the lung using test lines, but cannot be directly interpreted as a measure of alveolar size. MLI can be most directly interpreted as the mean free distance between gas exchange surfaces in the acinar airway complex [1]. Importantly, the use of MLI in estimation of the structural substrate of lung function is only applicable when considered a function of the total volume and surface area of the lung due to the lung’s structural complexity and complex inflational behavior [6]. Clinically, MLI has been associated with emphysematous changes to the lung and lung injury related to smoke and PM exposure [7, 8].

The degree of morphologic changes in affected lung may be accomplished using stratified random field selection and subsequent qualitative morphometric and pathologic analysis, such as the Destructive Index [3]; however, such methods may introduce unnecessary bias, so quantitative analyses such as MLI are preferred.

Importantly, there are several limitations in regards to MLI measures. There is no gold standard for the assessment of MLI [9]. MLI can be estimated by point or intersect-counting (superimposing a test line on a field and counting the number of times the line intercepts an alveolar septum, then using appropriate formulae) or directly measured by superimposing a test line on an image and measuring the distance along the line between consecutive intersections with alveolar septa [3]. Both intercept-counting and direct measurement, when performed manually, may take days to process even a few specimens. Additionally, there are no accepted values pertaining to sampling breadth, with the number of chords assessed per lung ranging from hundreds to thousands [3, 10–12].

Some of these concerns can be addressed using custom-built software that can achieve fully automated measuring processes; however, these are often technically intensive and rife with potential error, including challenges related to the true identification of non-alveolar structures, and a tendency of automated methods to underestimate MLI compared to manual measurements, as reported by Sallon et al., as well as Langston and Thurlbeck [13, 14]. In addition, this software is not made freely available to the public; therefore, there is need for a free, open-source, semi-automated solution. Such a method would allow a degree of supervision to control for spontaneous error and produce reliable results. By adapting the system of test lines traditionally used in manual quantitative assessment for implementation in a semi-automated method, our objective is to produce reliable measures of MLI. We utilized a modified version of our semi-automated measure of MLI in our recent publication [8]. Furthermore, the method was utilized in several abstracts and presentations [15–21]. Additionally, we developed a guide to determine the optimal number of chords for measurement to achieve accurate estimation of MLI per field of view. Our method relies on free, open-source software, does not require more than basic knowledge of computers, and, with the use of provided macros and plugin, has the potential to process hundreds of images in seconds (s) [22].

## Methods

### Computer-generated data

To ensure adequate performance, our semi-automated method was used to quantify MLI of fields of computer-generated circles of known dimensions. Fields of randomly placed, uniform-radius (r) circles were generated using a brute-force algorithm that ensured no circles overlapped. Measured MLI values (MLI_measured_) were compared to theoretical MLI values (MLI_actual_) via one-sample t-test. The effect on accuracy of the number of chords sampled was estimated using a repeated random subsampling procedure implemented in RStudio (R 3.4.3, R-Project), followed by receiver-operator-characteristic (ROC) curve analysis (IBM SPSS 23) to determine an optimal number of chords to measure per image [23, 24]. A reliability analysis (IBM SPSS 23) was performed on MLI_measured_ using a two-way mixed effects model to determine reliability of the measurements.

Additionally, fields of randomly placed, random-radius (5–50 pixels), non-overlapping circles were generated using a brute-force algorithm, and MLI_measured_ was assessed using the same procedure as above to estimate an optimal number of chords to use per-image.

### Murine PM exposure model and MLI measurement

Female wild-type (WT) C57Bl/6 mice(Jackson) > 12 weeks-old with free access to food/water and 12-hour (h) light/dark cycles were used [8, 25, 26]. World Trade Center-Particulate Matter < 53 μm (WTC-PM_53_) (200 μg) in sterile phosphate-buffered saline (PBS) or equal volume of PBS (controls) was aspirated under isoflurane-anesthesia (*n* = 3 per group), as previously described [8, 25, 27, 28]. PM dose was used within the measured concentration at the 9/11 debris pile [29]. After 24-h, mice were anesthetized by intraperitoneal Ketamine/Xylazine (100/10 mg/ml; 0.11 ml/10 g, Troy), and were sacrificed by exsanguination. Lungs were fixed in situ with 4% paraformaldehyde (Sigma) at 25 cmH_2_O and in 70% ethanol (4 °C). Lungs were processed through a series of graded ethanol, from 70 to 100%, xylene, and paraffin (Leica Peloris). Lungs were sectioned at 5 μm onto charged slides using a rotary microtome as previously described [25, 30]. Optimal lung sampling has been discussed in several studies [1, 10, 31, 32]. Longitudinal coronal sections were cut on a plane to include mainstem bronchi [25]. Lung sections were stained with hematoxylin and eosin (H&E) for primary assessment of structural architecture [25, 30]. The stained slides were digitally scanned using Slidepath (Leica).

To select fields for analysis, a grid of squares (520 μm × 520 μm) was laid over the entire lung section in Slidepath (Leica) at 2X magnification. Fields were chosen systematically to optimize unbiased random sampling of the section by selecting every fifth field from left to right, starting from the top left of the grid [1]. Fields that were not entirely tissue, such as those at the edge of the lung, were excluded. This method was repeated until 10 fields were selected according to previously published guidelines [1]. Each image was cropped at 20X magnification and treated as a separate image for the purpose of MLI quantification. All murine experiments were performed under approval of New York University-Institutional Animal Care and Use Committee-Protocol s16–00447 [8, 25, 27, 28].

### Automated assessment of MLI

**Fig. 1:**
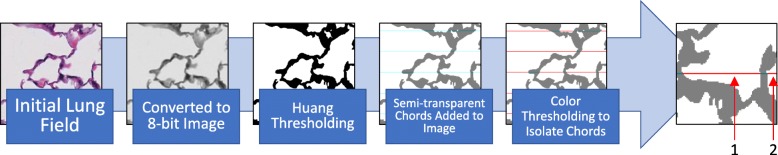
Process Schematic. Semi-automated Measure of Mean Linear Intercept. The initial lung field is converted to an 8-bit image, then Huang thresholding binarizes the image to denote airspace and lung. Semi-transparent test lines are then added to the image, and color thresholding is used to isolate discrete chords based on pixel color. The chord indicated by arrow 1 represents a genuine chord, whereas the chord indicated by arrow 2 represents a chord that would be excluded as a false intercept (its true length is uncertain)

On average, 582 chords per image and 5820 chords per lung were assessed in the previous study [3, 8, 10–12]. Using ImageJ macros on a Mac with 4.2 GHz Intel 4-core i7 and 32 GB of 2400 MHz DDR4 RAM running OS 10.12.6 (Sierra), each image was binarized and overlaid with 15 semi-transparent, horizontal test lines (opacity = 50%) spaced 35.4 μm apart. Discrete chords traversing alveolar septa, isolated based on pixel color, were measured, Fig. 1 (ImageJ). This process was repeated for vertical test lines [1, 5, 10, 11, 33]. Chord lengths were pooled per exposure group and analyzed as averages using independent-samples t-tests (IBM SPSS 23).

**Protocol for Semi-Automated Quantification of Mean Linear Intercept** (Detailed Protocol can be found in Additional file 1, and an ImageJ plugin is available free for academic research use at https://med.nyu.edu/nolanlab)

1. Creating test lines
  1.1. Horizontal test lines
    1.1.1. Open source image in Fiji
    1.1.2. Set parameters
    1.1.3. Set width and height
    1.1.4. Analyze
    1.1.5. Overlay image and save as a .Tif file
  1.2. Repeat for vertical test lines
2. Processing lung fields
  2.1. Thresholding
    2.1.1. Open lung image in Fiji
    2.1.2. Convert the image to 8-bit image
    2.1.3. Apply Huang thresholding
  2.2. Isolating chords
    2.2.1. Add test line image at 50% opacity to lung field image using the overlay function
    2.2.2. Flatten the image
    2.2.3. Isolate chords to be measured using the color thresholding function
3. Measuring chords
  3.1. Set measurement parameters
  3.2. Measure and record chord lengths
    3.2.1. Use the analyze particles function to measure isolated chords
    3.2.2. Chord measurements open in the results window and can be exported to excel or another appropriate software for analysis
  3.3. Repeat steps 2–3.2 using vertical test lines to isolate and measure vertical chords
4. Speeding up processing time
  4.1. Use Fiji macros to automate steps 2–3
    4.1.1. Apply Additional file 2 to the images to isolate chords
    4.1.2. Apply Additional file 4 to the output images of step 4.1.1 to measure chords
    4.1.3. Export chord measurements
    4.1.4. Repeat steps 4.1.1–4.1.3 for the vertical test line image, using Additional file 3 in step 4.1.1
  4.2. ImageJ plugin that performs all steps also available

## Results

### Virtual data set creation and MLI assessment

**Fig. 2:**
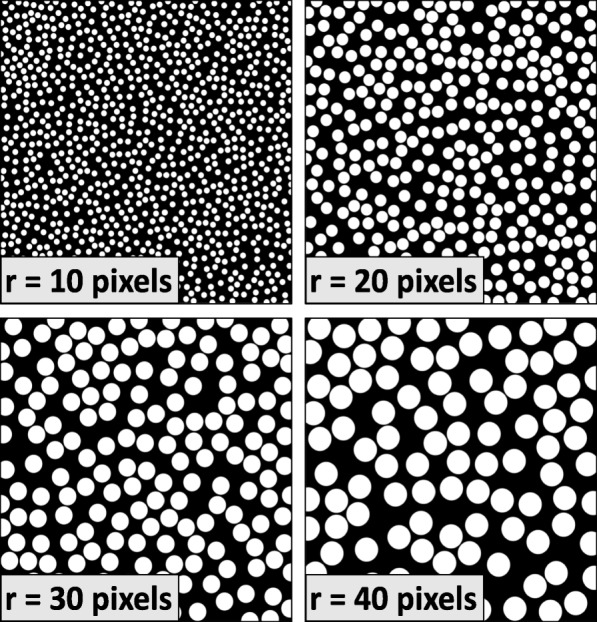
Samples of Computer-generated Images. Ten sets of 10 fields of randomly-placed, uniform-radius circles were measured. Radii of circles ranged from 5 to 50 pixels, in increments of 5 pixels

Using the previously discussed brute-force algorithm, sets of images (1000 pixels × 1000 pixels) were generated, Fig. 2. These images were stratified into 10 sets of 10 images, with each set containing 1 image from each r stratum (radii ranged from 5 to 50 pixels by 5-pixel increments). Our method was applied to these images to obtain MLI_measured_. MLI_actual_ is $\frac{r\pi}{2}$ for a circle of radius *r* by Cauchy’s formula for convex bodies in **R**^2^ [34].

Additionally, MLI_measured_ was assessed via our method for 10 images (1000 pixels × 1000 pixels) of random-radius circles generated as described in the methods to approximate heterogeneity observed in the lung. MLI_actual_ for these images was calculated per image as $\frac{\pi }{2}\frac{\sum \limits_i{r_i}^2}{\sum \limits_i{r}_i}$, where r_i_ is the radius of the i^th^ circle of an image.

### Optimization of number of chords to sample to accurately estimate MLI_actual_

**Fig. 3:**
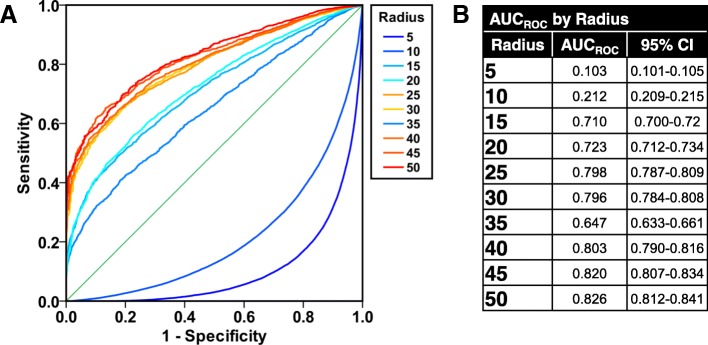
ROC Curves to Optimize the Number of Chords to Sample. **a** ROC curves were generated to test the sensitivity and specificity of number of chords in estimating MLI_actual_ per radius stratum. **b** AUC_ROC_ values and 95% confidence intervals as a classification performance measure

**Fig. 4:**
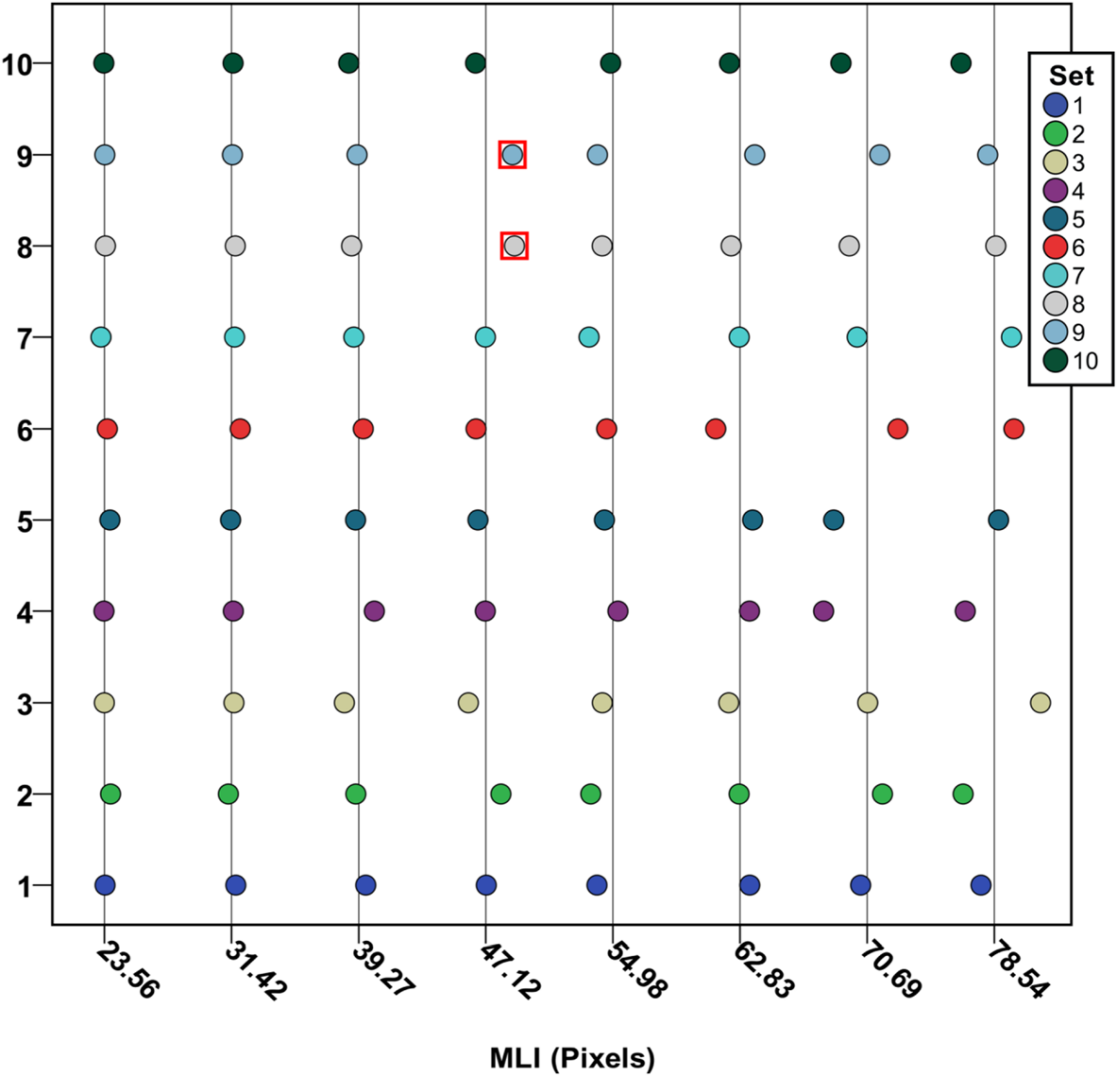
Dot Plot of MLI_measured_. Dot plot of intraclass correlation. We demonstrate high reliability with ICC = 0.9998. Vertical rule lines represent MLI_actual_. Represents significant deviation from MLI_actual_ by one-sample t-test, *p* < 0.05. Data for MLI_actual_ corresponding to radii of 5 or 10 pixels not shown due to poor AUC_ROC_ as discussed in Table 1

Application of the method yielded a data set of 118,716 chords measured across the computer-generated images of uniform-radius circles. A repeated random subsampling algorithm was implemented on this dataset to estimate MLI_measured_ using N randomly sampled chords from the pool of all chords measured per image (numbering N_max_); N was an integer on the interval [2, N_max_] and for each N, 5 random subsamples were used. The number of chords sampled per image was then analyzed via ROC curve to determine the ability of N chords to accurately estimate MLI_actual_ as *p* ≥ 0.05 by one-sample t-test, Fig. 3a. AUC_ROC_ is a nearly monotonic function of r stratum, Fig. 3b. This non-monotonicity is likely due to higher relative noise when measuring smaller radii circles. We then used Youden’s Index to estimate the optimal number of chords to sample to accurately measure MLI_actual_, Table 1. The optimal number of chords per r stratum was used to calculate MLI_measured_, Fig. 4. Finally, a reliability analysis was performed on MLI_measured_, stratified by r, using a two-way mixed effects model to determine the reliability of the measurements. We demonstrated excellent reliability, with ICC = 0.9998 and *p* < 0.0001, Fig. 4.

**Table 1:**
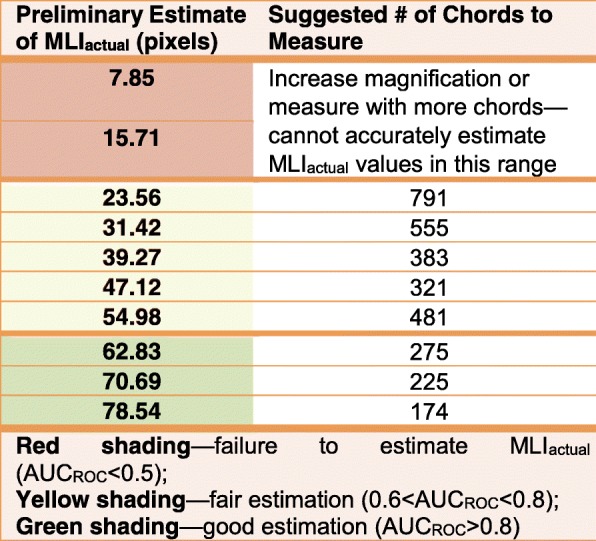
Optimal number of chords to estimate MLI_actual_

Red shading—failure to estimate MLI_actual_ (AUC_ROC_<0.5)

Yellow shading—fair estimation (0.6<AUC_ROC_<0.8)

Green shading—good estimation (AUC_ROC_>0.8)

The same procedure described above was applied to the images of random-radius circles, yielding a total of 12,981 chords measured. The optimal number of chords to accurately measure MLI was determined via ROC curve and Youden’s Index per-image. We show the optimal number of chords, AUC_ROC_, and MLI_actual_ per image, Table 2.

**Table 2:** Optimal number of chords to estimate MLI_actual_ in random-radius circle images

MLI_actual_	Optimal Number of Chords	AUC_ROC_(95% CI)
31.55	415	0.822 (0.789–0.855)
31.82	397	0.799 (0.769–0.829)
31.81	464	0.824 (0.795–0.853)
30.96	483	0.763 (0.733–0.793)
31.93	474	0.810 (0.780–0.839)
32.73	445	0.841 (0.814–0.868)
31.69	500	0.811 (0.781–0.840)
29.94	497	0.749 (0.716–0.782)
31.07	501	0.819 (0.784–0.853)
28.66	363	0.830 (0.801–0.860)

### Method optimization

Implementation of the method was optimized for speed as described in section 4 of the detailed protocol. Using Fiji macros, the time to execute this procedure per image was roughly 0.03 s.

### Real-world application

**Fig. 5:**
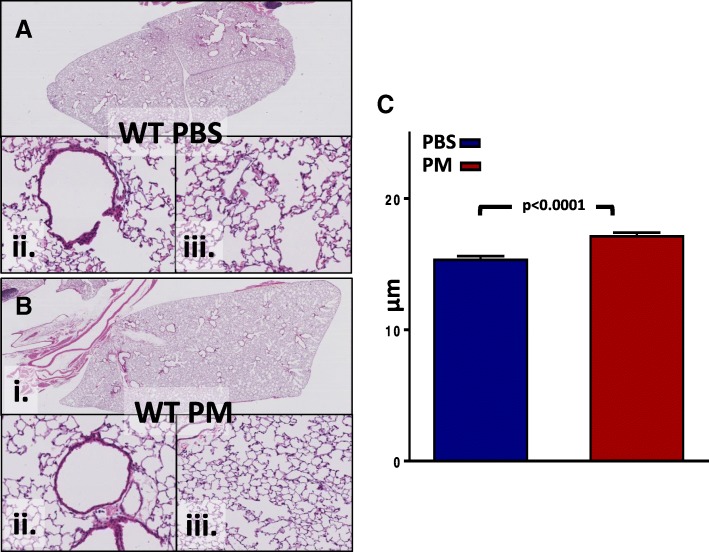
Application **a**. WT PBS **b**. WT PM Panel (i) at 2X, while (ii) & (iii) at 20X magnification. Mean free distance of gas exchange surfaces within the acinar airway complex **c**. After 24-h, WT mice exposed to PM had significantly increased MLI compared to WT PBS controls

In the murine model of WTC-PM exposure, a significant increase in MLI was observed in wild type, WTC-PM-exposed mice compared to PBS-exposed controls after 24-h, *p* < 0.05, Fig. 5 [8].

## Discussion

Overall, there is a significant need for a free method of lung histology quantification. MLI is a common measure used in pulmonary research; however, MLI measures have been difficult to obtain due to either the need for time consuming hand-measurements under a microscope via repeated superposition of a test line, or the need for proprietary software that may not be readily available. Despite availability of image-processing software, new computational tools remain under-exploited for application to the measurement of MLI. Our goal was to identify, optimize, and make readily available a method to measure MLI using freely available software. In addition, we wanted a method that would allow for an assessment of a significant amount of the lung.

Our method uses a novel combination of existing image processing functions that minimizes prerequisite, specialized computer-programming knowledge. While Fiji is used in this paper, any image processor with the ability to isolate test lines from background, overlay a semi-transparent layer on another image, and select non-contiguous pixels based on color, could also be used.

Processing one image (1000 pixels × 1000 pixels) in roughly 0.03 s, our method is faster than pre-existing methods. If performed manually, these measures take as long as 3 min per image (1200 pixels × 1600 pixels).(13) Our method compares favorably with fully automated methods, the most recent of which has been reported to take 0.6–0.9 s per image (1200 pixels × 1600 pixels).(13) Given algorithm design, it is unlikely that the observed differences in processing time are due in majority to image size differences.

Finally, we present a guide to how many chords to measure per field of view, given an estimation of MLI for that field. This can be used in an iterative process to tune the number of test lines used per image to best estimate MLI for that image. These numerical guidelines can be used to determine a suitably high magnification for image analysis, but, importantly, should only be applied to measures in pixel units.

There are no accepted values for the amount of lung to sample. While we present no guidelines on how much lung to sample, it would not be difficult to assess the entire lung specimen using a block-processing algorithm. According to the “do more less well” principle, it is cost ineffective to expend significant resources to ensure the genuineness of each intercept [35]. With the emphasis of our method on speed, there is effectively no cost to achieving high local accuracy while sampling a larger percentage of the lung. The paradigm of whole-slide processing gives new meaning to the “do more less well” principle. High accuracy per image/block (high local accuracy) is the only parameter of interest in a discussion of accurately measuring MLI for the whole lung. In fact, the distribution of chord lengths could be mapped across the lung, allowing a more comprehensive characterization of pathology than just a single MLI value per specimen. Our guide is meant to answer the question of how much is enough to ensure local accuracy and to control for image processing methods used.

As for commentary on usage of the guide: if MLI is small, it is probably worth using higher magnification to achieve better resolving power, as at this size of MLI, non-random effects due to thresholding methods become non-negligible, Table 1. We consistently observed this non-random effect in our set of computer-generated images and this effect is why we observe AUC_ROC_ < 0.5 for the 5- and 10-pixel radius strata. Also, the proposed numbers of chords are overestimates compared to what would be necessary when measuring lung morphology. This effect is due to the negative skew of the distribution of individual chord lengths in uniform-radius fields compared to the positive skew of the same distribution for random-radius circles and actual lung. In random-radius circles and lung tissue, longer chords make up a larger percentage of the total chords measured, whereas the upper bound of chord lengths in uniform-radius circle images is set by the diameter of the circle, and indicates that a higher portion of chord lengths will be in the 5- or 10-pixel range. This distributional phenomenon is reflected by the lower optimal number of chords found necessary when measuring random-radius circles, Table 2.

There are a number of limitations of the parameter MLI which have been discussed extensively elsewhere [1, 3]. An alternative proposed by Parameswaran et al. can be readily calculated from the distribution of individual chords yielded by the present method [2]. In this article, we present only a method of estimation of MLI. Importantly, we optimize the use of a previously established system of test lines to minimize time and tedium; therefore, the limitations of the test-line system are beyond the scope of this paper. For a thorough discussion of these limitations, the reader is referred to the following articles [3, 9]. Finally, in application to a murine model, we acknowledge that there are a number of fixation method parameters and lung sampling procedures that can be optimized for different purposes. These considerations are outside the scope of this paper and are discussed elsewhere [1].

Our method has several limitations and a number of application parameters are important to consider. These limitations include the image resolution—the resolving power of the method is 1 pixel; therefore, it is important to use images under sufficiently high magnification, where the 1-pixel resolving power is negligible compared to the average length of the chords to be measured.

Fully automated methods consistently underestimate MLI compared to manual measures [13, 14]. We do not know how our method compares to manual measurements in terms of under- or overestimation of MLI. Additionally, we present no method of eliminating blood vessels and other non-alveolar structures present on histology. There is arguably little worth in pursuing such structure recognition, due to its high computational cost and the “do more less well” principle as previously discussed. Because hundreds of chords can be measured per image, chord measurements produced by these structures are negligible.

## Conclusion

In conclusion, our semi-automated MLI measurement method allows for quantification of a large area of lung, and relies on freely available software. The method produced reliable, accurate measurements and compares favorably with other methods in terms of computational requirements and speed. We successfully applied a modified version of the method to murine histology to show anatomical changes. The modification was simply in the sense of integration of multiple software platforms; the method was later streamlined to use only one platform. Given the nature of the method, it could be easily adapted to make serial measures of other types of tissue. Future applications include implementation as a premier method of histological quantification in a laboratory setting.

## Additional files

Additional file 1: Detailed Protocol for Semi-Automated Quantification of Mean Linear Intercept. Step-by-step description of the method. (DOCX 18 kb)

Additional file 2: Macro which binarizes the images and isolates *horizontal* chords. (TXT 1 kb)

Additional file 3: Macro which binarizes the images and isolates *vertical* chords. (TXT 1 kb)

Additional file 4: Macro which measures chords (This can measure horizontal or vertical chords). (TXT 413 bytes)

## Data Availability

The datasets used and/or analyzed during the current study are available from the corresponding author on reasonable request. We have also made available in our **online supplementary file**, pre-written macros in plain text format for Steps 2 and 3.

## Abbreviations

AUC: Area Under the Curve
CDC: Centers for Disease Control
CI: Confidence Interval
cmH_2_O: Centimeters of Water
h: Hour
IBM: International Business Machine
ICC: Intraclass Correlation Coefficient
MLI: Mean Linear Intercept
NHLBI: National Heart, Lung, and Blood Institute
NIOSH: National Institute for Occupational Safety and Health
PBS: Phosphate-buffered Saline
PM: Particulate Matter
r: Radius
ROC: Receiver Operator Characteristic
s: Seconds
TIFF: Tagged Image File Format
WTC: World Trade Center
WTC-PM_53_: World Trade Center-Particulate Matter < 53 μm
μm: Micrometers
