# Supplementary material for: Quantitative lung morphology: semi-automated measurement of mean linear intercept
Source: BMC Pulm Med. 2019 Nov 9;19:206. doi: 10.1186/s12890-019-0915-6 (PMC6842138; doi:10.1186/s12890-019-0915-6)
Supplement: Supplementary file 1 — Detailed Protocol for Semi-Automated Quantification of Mean Linear Intercept. Step-by-step description of the method. (DOCX 18 kb) [file 12890_2019_915_MOESM1_ESM.docx]

**Detailed Protocol for Semi-Automated Quantification of Mean Linear Intercept**

**1. Creating Test Lines**

- 1. For Horizontal Test Lines
     1. In Fiji (an open source image processing package for use with ImageJ), create a new image by selecting File> New> Image…
     2. Choose the following parameters: Type =RGB; Fill With =White; Slices= 1
     3. Set Width and Height to match the dimensions of the images to be analyzed, and select OK.
     4. Next, select Analyze> Tools> Grid, and choose the following parameters: Grid Type = Horizontal Lines; Area per Point should be adjusted to provide the desired number of test lines; leave Bold, Center grid on image, and Random offset unchecked.
     5. Finally, select Image> Overlay> Flatten, and then save as a Tiff file with the name “horizontal.tif”.^[[1]](#footnote-1)^ Note: Naming this file “horizontal.tif” is crucial to the provided macros functioning correctly.
  2. **For Vertical Test Lines**
     1. If the width and height of the images to be analyzed are identical, simply select Image> Transform> Rotate 90 Degrees Right and then save as a Tiff with the name “vertical.tif”
     2. If the width and the height of the images to be analyzed differs, follow the same steps as section 1.1.1. However, when creating a new image, switch the Width and Height dimensions. After Flattening the image, select Image> Transform> Rotate 90 Degrees Right, then save as a Tiff file with the name “vertical.tif”. Note: To maintain the same spacing between horizontal and vertical test lines, keep the Area per Point value the same.

1. **Processing Lung Fields**
   1. **Thresholding**
      1. Open lung image in Fiji.
      2. Convert the image to an 8-bit image by selecting Image> Type> 8-bit.
      3. Next, select Adjust> Threshold from the Image dropdown menu. Change the threshold method to Huang and then click Apply.
   2. **Isolating Chords**
      1. Open horizontal test line image file.
      2. Select the window of the lung field image.
      3. Select Image> Overlay> Add Image… and select the following options: Image to add = *select your test line image here*; X location = 0; Y location = 0; Opacity = 50; Zero transparent should be left unchecked. Click “OK” to add the test line image to the lung field image.
      4. Select Image> Overlay> Flatten.
      5. Next, select Image> Adjust> Color Threshold… Ensure the following options are selected: Thresholding method = Default; Threshold color = Red; Color space = HSB; the “Pass” boxes corresponding to Hue, Saturation, and Brightness should be checked; Dark background should be left unchecked. Adjust the “Hue” sliders to surround the narrow peak around the hue of the test lines. An interval of roughly [120, 140] is sufficient for cyan test lines. Set the intervals for Saturation to [0, 255] by adjusting the corresponding sliders. Adjust the “Brightness” sliders to so that only the chords to be measured are covered by the threshold mask (highlighted in red). We find the interval [140, 255] to be sufficient. Visually verify that the threshold mask correctly masks only the chords.
2. **Measuring Chords.**
   1. **Set Measurement Parameters**
      1. Choose Analyze> Set Measurements… Only the following options need be selected: Bounding rectangle, Limit to threshold, and Display label. Click Ok to apply.
   2. **Measure and Record Chord Lengths**
      1. Then select Analyze> Analyze Particles… and set the parameters to: Size = 0-infintiy; Circularity = 0.00-1.00; Show = Nothing; and only select the following check boxes: Display results and exclude on edges.
      2. Click Ok. The chord measurements open in the Results window and can be exported to Excel or another appropriate software for analysis.
   3. Repeat steps 2-3.2 using vertical test lines to isolate and measure vertical chords.
3. **Speeding Up Processing Time**
   1. In Fiji, macros can be used to automate steps 2 and 3 and therefore decrease processing time. No knowledge of the language used in Fiji macros is required. Pre-written macros have been provided in plain text format in the online supplementary material. There are three macros: Additional file 2 binarizes the images and isolates *horizontal* chords; Additional file 3 binarizes the images and isolates *vertical* chords; and Additional file 4 measures chords (Additional file 4 can measure horizontal or vertical chords).
      1. Additional file 2: To use this macro, first open the horizontal test line image, and then select Process> Batch> Macro… Choose appropriate Input and Output folders (The Input folder should contain only the images to be measured and the Output folder should be empty), and set Output format to TIFF. Click the “Open…” button, open Additional file 2, and click “Process” to isolate chords.
      2. Additional file 4: Next select Process> Batch> Macro… and set the Input folder for this step to be the Output folder populated by step 4.1.1. Choose an appropriate Output folder for this step (any empty folder will do) and set Output format to TIFF. Click the “Open…” button, open Additional file 4, and click “Process…” to measure chords.
      3. The chord measurements open in the Results window and can be exported as a comma-separated values file.
      4. Repeat steps 4.1.1-4.1.3 for the vertical test line image, using Additional file 3 in step 4.1.1.
   2. Additionally, we have developed an ImageJ plugin to perform these functions and made it available free for academic research use at <https://med.nyu.edu/nolanlab>.

1. It is standard practice to save all image files in a high bit-depth tiff file format to avoid effects that other file formats, such as jpeg, would have on image integrity. [↑](#footnote-ref-1)
